# Dyke-Davidoff-Masson Syndrome Underlying Hemiplegic Cerebral Palsy in an Adolescent: A Case Report

**DOI:** 10.7759/cureus.112746

**Published:** 2026-07-15

**Authors:** Jarrod Jie Rong Chua

**Affiliations:** 1 Lee Kong Chian School of Medicine, Nanyang Technological University, Singapore, SGP

**Keywords:** a case report, cerebral palsy (cp), computed tomography (ct ), dyke-davidoff-masson syndrome (ddms), neurology and pediatric neurology, rare neurological disorder, spastic hemiplegia

## Abstract

Dyke-Davidoff-Masson syndrome (DDMS) is a rare clinicoradiological syndrome involving unilateral cerebral hemiatrophy with compensatory calvarial changes. It typically presents with seizures, contralateral hemiparesis or hemiplegia, and developmental delay. The clinical manifestations may overlap with those of hemiplegic cerebral palsy, potentially delaying recognition of the underlying structural diagnosis.

We report a 14-year-old male with longstanding right hemiplegic cerebral palsy and focal epilepsy who presented following a bicycle-related injury. During admission, non-contrast computed tomography of the brain was done to exclude intracranial pathology due to complaints of persistent headache. Imaging demonstrated extensive left frontoparietal encephalomalacia involving the middle cerebral artery territory, ex vacuo dilatation of the left lateral ventricle, ipsilateral Wallerian degeneration, and calvarial thickening, consistent with DDMS. No acute intracranial abnormality was identified. The patient recovered and was subsequently discharged with continued anti-seizure medications and multidisciplinary outpatient follow-up.

This case highlights the importance of considering DDMS in patients with hemiplegic cerebral palsy and epilepsy. Recognition of DDMS provides an anatomical explanation for the patient's neurological phenotype, enhances understanding of the underlying cerebral injury, and supports long-term multidisciplinary management.

## Introduction

Dyke-Davidoff-Masson syndrome (DDMS) is a rare clinicoradiological condition that was first described in 1933 by Dyke, Davidoff, and Masson [1]. To date, <100 cases have been reported worldwide. DDMS is characterised by seizures, contralateral hemiparesis or hemiplegia, facial asymmetry, developmental delay, intellectual disability, and behavioural difficulties [2]. Typical radiological features include unilateral cerebral hemiatrophy, ipsilateral ventricular dilatation, widened cortical sulci, calvarial thickening, and hyperpneumatisation of the ipsilateral frontal sinus or mastoid air cells [3].

The condition may be congenital or acquired. Congenital types are thought to result from intrauterine cerebral vascular or developmental insults, while acquired types may follow from perinatal or early childhood events such as trauma, infection, vascular insult, or prolonged seizures [4]. As the clinical manifestations are non-specific and overlap with several neurological disorders, recognition of DDMS may be delayed until characteristic neuroimaging is obtained.

The clinical features of DDMS may overlap with hemiplegic cerebral palsy. In such patients, cerebral palsy describes the non-progressive motor impairment, while DDMS explains the underlying anatomical abnormality. Herein, we present a case of DDMS in an adolescent with right hemiplegic cerebral palsy and focal epilepsy who presented with recurrent breakthrough seizures. This case highlights the importance of correlating clinical features with neuroimaging to recognise DDMS as the structural basis of the patient's neurological phenotype.

## Case presentation

A 14-year-old male with a background of right hemiplegic cerebral palsy and focal epilepsy presented to the emergency department of a tertiary hospital following a bicycle-related injury. He sustained a closed right tibia and fibula fracture when his leg became caught in the bicycle, and he fell onto his right side. There was no reported head injury, handlebar injury, or seizure preceding the fall. The bicycle-related injury represented an acute event occurring many years after the onset of his neurological deficits and established diagnoses of hemiplegic cerebral palsy and epilepsy.

He had a known history of focal epilepsy and right hemiplegic cerebral palsy. His known seizure semiology consisted of head and eye deviation to the right, with some episodes associated with limb stiffening and jerking. He was under neurology follow-up and was taking controlled-release carbamazepine 200 mg twice daily and sodium valproate chrono 750 mg every morning. Dose escalation of carbamazepine had been considered in the event of further seizures.

The patient had previous admissions for breakthrough seizures one month ago. During one admission, he presented after several self-aborting episodes at school, each lasting approximately five minutes. The episodes were considered likely breakthrough seizures in the context of known focal epilepsy, with possible contributing factors, including upper respiratory tract infection and suboptimal anti-seizure medication adherence. His sodium valproate dose was subsequently increased during that admission.

He also had a history of learning and behavioural difficulties, with previous psychology or psychiatry input. Available documentation suggested possible borderline intellectual functioning and possible attention-deficit/hyperactivity symptoms. Psychosocial difficulties were also noted, including parent-child relationship difficulties and school stressors. Details were kept limited to preserve confidentiality and relevance to the neurological presentation.

Antenatal, perinatal, and early childhood histories were not available from the records reviewed. Therefore, it was not possible to determine whether the cerebral insult underlying the DDMS was congenital or acquired.

In the emergency department, the patient was managed with supportive care, neurological observation, and seizure monitoring. In view of his previous breakthrough seizures, underlying neurological comorbidities, and psychosocial complexities, he was admitted under paediatric medicine for further monitoring.

Examination findings

On admission, the patient was alert and haemodynamically stable. Examination was significant for a right lower limb injury consistent with a closed tibia and fibula fracture. Motor examination was consistent with his known right spastic hemiplegic cerebral palsy. The right upper limb was hypertonic and held in a flexed, mildly pronated posture, while the right lower limb demonstrated mild hypertonia. All four limbs moved against gravity, and there were no new focal neurological deficits compared with his baseline examination.

During admission, he developed a persistent headache overnight and was reassessed. He remained alert with a Glasgow Coma Scale score of 15, and no reduction in consciousness was documented. There was no new focal neurological deficit beyond his baseline right hemiplegic cerebral palsy.

Assessment

In view of the persistent headache and his neurological history of cerebral palsy and focal epilepsy, laboratory investigations and a non-contrast computed tomography (CT) of the brain were performed to exclude acute intracranial pathology. Full blood count, renal function, serum electrolytes, calcium, magnesium, and phosphate were normal. Blood culture showed no bacterial growth.

**Figure 1:**
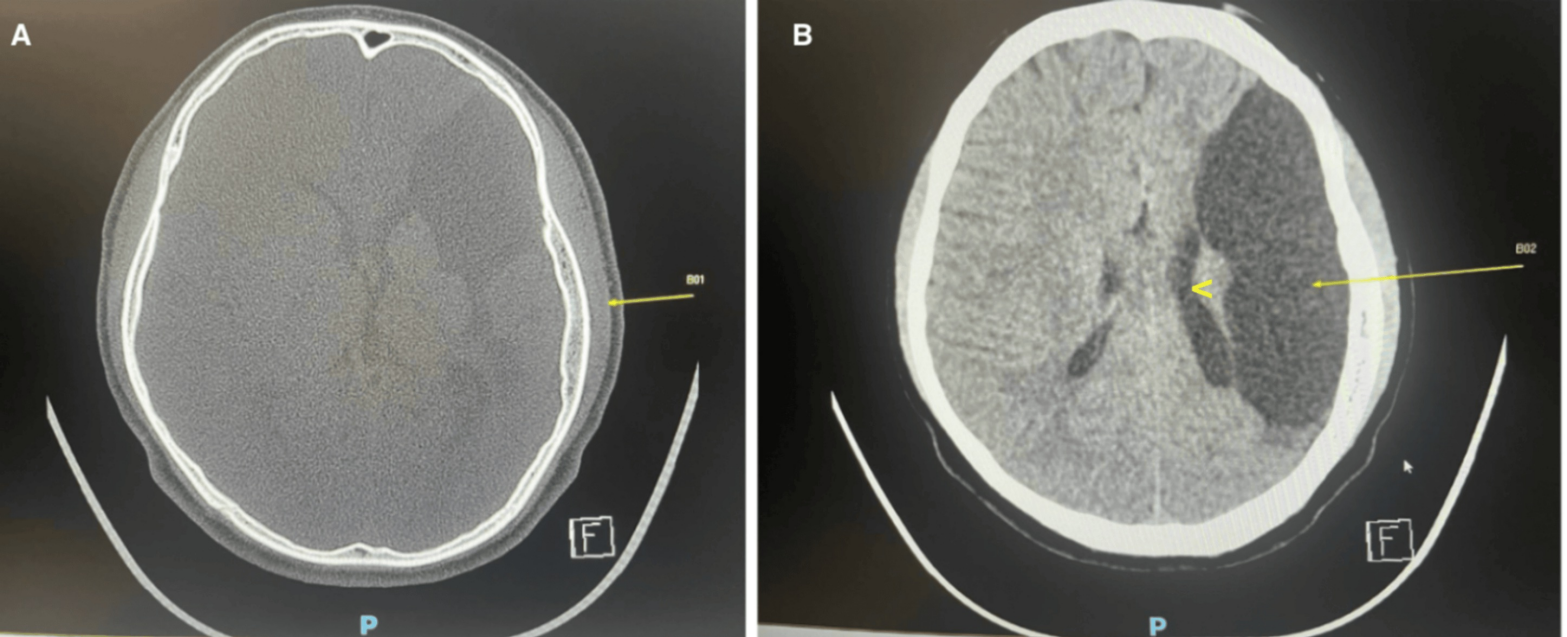
Non-contrast CT of the brain demonstrating features of Dyke-Davidoff-Masson syndrome. (A) Axial CT image demonstrating left cerebral hemiatrophy with ipsilateral calvarial thickening. (B) Axial CT image at the level of the lateral ventricles demonstrating extensive left frontoparietal encephalomalacia (arrow) involving left middle cerebral artery territory with associated ex vacuo dilatation of the left lateral ventricle (arrowhead).

Non-contrast CT of the brain (Figure 1) demonstrated characteristic chronic left cerebral abnormalities. Figure 1A shows ipsilateral calvarial thickening, while Figure 1B shows extensive left frontoparietal encephalomalacia involving the middle cerebral artery (MCA) territory with associated ex vacuo dilatation of the left lateral ventricle and ipsilateral Wallerian degeneration. Mild atrophy of the left basal ganglia, thalamus, and temporal lobe was also noted. No acute territorial infarction, intracranial haemorrhage, hydrocephalus, or midline shift was identified.

These radiological findings, in conjunction with the patient's longstanding contralateral spastic hemiplegia and focal epilepsy, were considered compatible with the clinicoradiological features of DDMS. The encephalomalacia likely represents the sequelae of a left MCA territory insult sustained during early brain development.

Although hyperpneumatisation of the frontal sinus and mastoid air cells is classically described in DDMS, these features were not reported in our patient [5]. Nevertheless, the presence of left cerebral hemiatrophy, ex vacuo ventricular enlargement, and ipsilateral calvarial thickening supported the impression of DDMS.

The patient’s headache symptoms improved with conservative management, and the patient was subsequently discharged home on existing sodium valproate and carbamazepine. The patient continues to follow up with paediatric neurology and psychiatry and receives rehabilitation, physiotherapy, and occupational therapy.

## Discussion

This case illustrates the close relationship between DDMS and hemiplegic cerebral palsy. Cerebral palsy is a clinical syndrome characterised by permanent, non-progressive disorders of movement, tone, and posture resulting from injury to the developing brain, whereas DDMS is a radiological diagnosis describing structural changes following an early cerebral insult [6]. Both conditions may therefore represent different manifestations of the same pathological process. In our patient, the diagnosis of right hemiplegic cerebral palsy had been established in early childhood clinically. However, neuroimaging subsequently demonstrated unilateral cerebral volume loss with compensatory cranial changes consistent with DDMS, providing an anatomical explanation for his motor impairment and epilepsy [3]. However, in the absence of early-life clinical records and MRI, these findings should be interpreted cautiously, as they are not entirely specific and may also be seen following other causes of early unilateral cerebral injury. This distinction is clinically important because recognising the underlying structural diagnosis improves understanding of the patient's neurological phenotype and assists with long-term prognostication and epilepsy management.

Neuroimaging findings

The extensive left MCA territory encephalomalacia, ex vacuo ventricular dilatation, and ipsilateral calvarial thickening are consistent with chronic cerebral injury sustained early in brain development [7]. These findings provide a plausible structural explanation of the patient's contralateral spastic hemiplegia and focal epilepsy. While the encephalomalacia likely reflects a remote left MCA territory insult, the combination of characteristic radiological findings together with the clinical phenotype is consistent with the clinicoradiological features of DDMS rather than chronic infarction alone. Although frontal sinus hyperpneumatisation was absent, this feature is not universally present and should not preclude the diagnosis.

Differential diagnoses

Sturge-Weber syndrome was considered but was unlikely because our patient lacked facial capillary malformations, leptomeningeal angiomas, and intracranial calcifications. Other manifestations include diffuse choroidal vascular malformation, loss of vision, seizures, hemiparesis, hemiplegia, developmental delays, cerebral atrophy, and calcifications. [8] Rasmussen encephalitis was also considered unlikely because it is typically associated with progressive neurological deterioration and intractable seizures and does not show calvarial changes. [9]. A remote MCA territory infarction is an important differential diagnosis because unilateral encephalomalacia and contralateral neurological deficits may be seen. However, DDMS is a clinicoradiological syndrome resulting from an early cerebral insult rather than a specific aetiology. In our patient, the longstanding history of hemiplegic cerebral palsy and epilepsy, together with unilateral cerebral hemiatrophy, ex vacuo ventricular dilatation, and ipsilateral calvarial thickening, raised the possibility of DDMS. Other differential diagnoses include unilateral megalencephaly, Silver syndrome, and Fishman syndrome.

EEG would have helped strengthen the evaluation of seizure semiology and long-term epilepsy management. However, the absence of EEG findings in this case does not exclude DDMS, as the diagnosis is primarily supported by clinical features and neuroimaging.

The management of DDMS is largely supportive. Anti-seizure medications such as sodium valproate, carbamazepine, and lamotrigine are prescribed with the aim of seizure control. Some studies have shown that combination therapy involving two or more drugs may be used. In Hamid et al. (2022), despite sodium valproate being administered with its dosage increased to 25 mg/kg/d, the partial seizures persisted [10]. Carbamazepine was then added, and the frequency of seizures decreased. A multidisciplinary team involving physiotherapy, speech therapy and occupational therapy is crucial in the long-term management of the patient. In selected patients with drug-resistant epilepsy, surgical options such as hemispherectomy may be considered [11].

Clinical significance

Several case reports have described DDMS in children presenting with refractory epilepsy or hemiplegia. However, reports describing DDMS in adolescents previously diagnosed with hemiplegic cerebral palsy remain limited. Our case emphasises that DDMS may remain unrecognised until adolescence, when neuroimaging is performed for unrelated clinical indications.

While it does not alter the diagnosis of cerebral palsy, this case is clinically relevant. It provides a more complete understanding of the underlying neurological phenotype, guides seizure management, and supports long-term multidisciplinary care planning.

Limitations

This case report has several limitations. Early-life medical records, including birth history, neonatal history, and early neurodevelopmental documentation, were not available because the patient’s earlier health records were from another country. Consequently, the timing of the cerebral insult could not be established, limiting our ability to distinguish congenital DDMS from acquired cerebral hemiatrophy. Second, no EEG report was available, limiting electroclinical correlation. MRI was not performed during this admission because the patient remained neurologically stable and CT findings were sufficient for acute management.

## Conclusions

DDMS should be taken into consideration in patients with hemiplegic cerebral palsy, epilepsy, and unilateral cerebral atrophy on neuroimaging. This case describes an adolescent with spastic hemiplegic cerebral palsy and focal epilepsy, who demonstrated characteristic neuroimaging findings of DDMS. Although the absence of early childhood records and MRI limited definitive characterisation of the underlying cerebral insult, this case highlights the importance of correlating clinical features with neuroimaging findings when evaluating patients with longstanding hemiplegic cerebral palsy. Additional reports and larger case series are needed to further improve understanding of the clinical spectrum and diagnosis of DDMS.
